# The Effect of Hybrid Barley in the Diets of Fattening Pigs on Pork Oxidative Stability Related to the Fatty Acid Profile

**DOI:** 10.3390/ani11072134

**Published:** 2021-07-19

**Authors:** Anna Szuba-Trznadel, Małgorzata Korzeniowska, Tomasz Hikawczuk, Bogusław Fuchs

**Affiliations:** 1 Department of Animal Nutrition and Feed Management, The Faculty of Biology and Animal Sciences, Wrocław University of Environmental and Life Sciences, Chełmońskiego 38 C, 51-630 Wrocław, Poland; tomasz.hikawczuk@gmail.com (T.H.); boguslaw.fuchs@upwr.edu.pl (B.F.); 2 Department of Functional Food Products Development, The Faculty of Biotechnology and Food Sciences, Wrocław University of Environmental and Life Sciences, Chełmońskiego 37, 51-630 Wrocław, Poland; malgorzata.korzeniowska@upwr.edu.pl

**Keywords:** fatteners, hybrid barley, pork quality, fatty acids, antioxidant status

## Abstract

**Simple Summary:**

The general objective of this study was to investigate the effect of hybrid barley and wheat in the diet of fattening pigs on the oxidative stability of pork related to the chemical composition, especially the fatty acid profile. The feeds used included 80% hybrid barley in group I, a mixture of 40% wheat and hybrid barley in group II, and 80% wheat in group III. The feeding trial lasted 78 days. Meat samples were taken from twelve carcasses, randomly selected, chosen from each experimental group. The meat analyses covered the physicochemical and sensory traits. The results showed a decrease in the palmitic acid concentration and an increase in the oleic acid concentration in the meat of fattening pigs fed a diet of 80% hybrid barley. The meat of these pigs was characterised by the best marbling, which was closely related to its juiciness after thermal processing and its final culinary quality. Moreover, meat samples from the hybrid barley fed pigs exhibited a reddish colour, before and after thermal processing. In summary, the addition of hybrid barley into the diets of pigs improved the quality of the culinary meat.

**Abstract:**

Feed determines the quality of pork meat, in which the composition of the fatty acid (FA) profile is one of the easiest to modify by the application of selected feed components. Barley grains are considered to have an impact on meat quality, including pork; however, there are still limited data on the use of hybrid barley in fattening pigs’ nutrition in relation to meat quality. The aim of this study was to determine the relation between meat quality, i.e., its oxidative stability, especially the FA profile, and fattening pigs’ diets with hybrid barley and/or wheat. In group I, hybrid barley (HB) composed 80% of the feed; in group II, a mixture of (40% each) wheat and barley was used; and in group III, wheat (W) composed 80% of the feed. Meat samples were taken from twelve randomly selected carcasses chosen from each group. The meat analyses covered the physicochemical and sensory traits. The results showed that the pork meat of fattening pigs fed fodder with 80% HB had decreased palmitic acid concentrations and increased oleic acid concentrations. The meat of these pigs was characterised by the best marbling, which was closely related to its juiciness after thermal processing and determined its final culinary quality. Moreover, the meat from these pigs exhibited a reddish colour, before and after thermal processing. In summary, the application of hybrid barley into pig nutrition improved the quality of the culinary meat.

## 1. Introduction

Achieving high-quality pork meat by including barley in pigs’ nutrition is a key aspect of meat processing [1,2]. Barley enhances the palatability and texture of meat due to relatively high contents of palmitic and stearic acids in fat tissue, as well as the beneficial effect of backfat on stability [3,4,5]. The backfat of barley-fed pigs is distinguished by desirable traits, such as marbling score and springiness. The tissue fat has similar assets. Such raw materials are favourable for producing high-quality shelf-stable meat products that can be stored for longer periods and are attractive to consumers. The lipids that compose the fat tissue of barley-fed fattening pigs are more resistant to rancidity as compared with fat obtained from animals provided with diets rich in polyunsaturated fatty acids (PUFA), for example, corn [6].

The meat of fattened pigs (usually fed with high amounts of grains and oilseeds) is characterised by a disadvantageous ratio of n-6 PUFA to n-3 PUFA (from 7:1 to 23:1) [7]. Therefore, taking into consideration long-lasting consumers’ health, the approach is to use dietary components in pig feed that are good sources of n-3 PUFA. According to many studies, the lipid fraction of animal products, especially in the case of fatty acid (FA) incidence and share, can be designed, to a certain extent, by modifying feed composition [8,9,10,11].

The general advantages of hybrid varieties of feed are higher and better yield stability. Moreover, hybrids are also characterized by better technological quality parameters. However, there are few studies on feeding hybrid barley to obtain modern high-quality pork meat. There is also a significant lack of data regarding its effect on lipid modification in meat, and in particular, the fatty acid profile in pork meat. The above reports prompted us to conduct this study aimed at determining whether hybrid barley used to feed fattening pigs could influence selected parameters of lipid metabolism in pork meat.

Desirable nutritional changes in the FA composition and share in pork are related to the concentration and profile of lipid fraction of the animal feed components and proportions. Variations in meat FA, through increased PUFA and reduced SFA, continues to be an important problem in terms of human health [12,13] and should also be implemented regarding animal health, and the quality of the products obtained from the animals. Therefore, it is important to produce meat characterised by a relatively low content of visible fat, but with very-high nutritional value as well as sufficient amounts of intramuscular fat that is necessary to assure proper meat quality (including juiciness and palatability), and consequently, to satisfy consumers’ expectations and needs.

The aim of this study was to use a hybrid barley in the feed of fattening pigs and to analyse the effect of the applied modifications on selected quality parameters of cut-up pork meat, including the oxidative stability determined by the FA profile and the chemical composition of the pork meat. In this study, during the fattening process, pigs were fed complete diets with high and medium contents of hybrid barley, which is not commonly found in the literature. Wheat grain was used as a reference material due to its very-high nutritional value and properties suitable for producing feed mixtures.

## 2. Materials and Methods

### 2.1. Animals, Diets, and Feeding

The feeding trial was performed at a specialized private pig farm after approval by the Local Ethical Review Committee for Animal Experiments in Wrocław, Poland (protocol no. 002/2019). One hundred and forty-four fattening pigs (Polish large white × Polish landrace crossbreds) were used in the experiment. The animals were selected based on an initial body weight of 55 kg, fattened for a period of 78 days, and finished when the animals reached weights of ca. 120 kg. Feeding was carried out in a semi ad libitum mode for the pigs kept in common pens, with 8 animals assigned to each pen, therefore, for each feed mixture group there were six pens. The animals had unlimited access to drinking water delivered by nipple drinkers.

The study plan assured a random relocation of fattening pigs into three groups, each with an equal number of animals (48 animals) but supplied with different types of especially designed (PT2 type) complete diets composed by various amounts/shares of hybrid barley (HB) and/or wheat (W). Hybrid varieties of barley Hyvido^TM^ (Syngenta Co., Warszawa, Poland) were used in the study. Animals assigned to group I were provided with a diet consisting of 80% HB, while pigs in group II received an equal (40% each) mixture of W and HB. Group III was the control group, which was fed a fodder with 80% W. The feed mixtures were given to animals in a free-flowing form that corresponded to their requirements [14]. In addition to grain ingredients (in the given proportions), the feed mixtures contained soybean meal and also fodder yeast, premixes with enzymes for swine and a mineral-vitamin mixture. The energy value of the feed mixtures ranged from 12.7 to 13.2 MJ·kg^−1^ (the highest value was in the group with the highest amount of wheat in the diet). As the wheat content increased, the amount of fat (from 11.7 to 20.9 g·kg^−1^) and protein (from 155 to 164.5 g·kg^−1^) also increased, while the fibre content (from 53.4 to 30.5 g·kg^−1^) decreased. The composition and nutritional value and detailed information regarding the chemical analyses of the diets were presented in an earlier study by Szuba-Trznadel et al. [15].

### 2.2. Meat and Fat Analyses

On the last day of the feeding experiment, all animals were slaughtered and dressed at an abattoir, in accordance with standard valid technology. Then, 12 randomly chosen carcasses per group (with a body weight corresponding to the average body weight for each studied group) were kept at chilling conditions of 2–4 °C for 24 h after slaughter to allow the proper turnover of muscle into meat, monitored by pH measurements (pH was analysed by placing a pH meter electrode Orion 3-Star pH Benchtop Meter (Thermo Fisher Scientific Inc., Waltham, MA, USA) directly into muscle tissue) at room temperature. The weight of cold carcasses ranged from 88.48 ± 9.10 kg to 89.36 ± 6.27 kg [15]. The meat samples (12 samples from each group) were collected post mortem (the middle part of the loin with bones and backfat, weighing ca. 1 kg each). In the laboratory before performing analyses, the meat was separated from the bones and backfat, and therefore the chemical analyses were conducted exclusively on the meat. The physicochemical analyses (WHC) were performed on fresh loin. The water holding capacity (WHC) of meat was analysed according to the Grau–Hamm method with modification by Szmańko [16]. Then, the meat was frozen (in temperature conditions of −18 °C) and used within one month. The samples were thawed, and chemical analyses and organoleptic characteristics were carried out. The proximate analysis of meat was performed according to AOAC [17] to evaluate the contents of basic nutrients. Moisture, fat, and protein contents were analysed in triplicate. Protein was analysed according to the PN-75/A-04018:1975 Polish Standard [18] on a Nitrogen Analyser Kjeltec TM 2300 (FOSS, Hilleroed, Denmark) to obtain the nitrogen content multiplied by 6.25 in order to determine the value of total crude protein. Ether extraction was applied to analyse the crude fat content, according to the PN-ISO 1444:2000 [19]. For the evaluation of the content of ash in the samples, first, dry matter was measured by evaporation of the free water during drying at 102 °C (PN-ISO 1442:2000 [20]), followed by complete sample incineration at 550 °C in a furnace for 16–18 h (PN-ISO 936:2000 [21]).

The composition and share of the specific individual FA in pork were determined by gas chromatography methods proceeded by lipid extraction using Folch solution (methanol/chloroform) [22]. Then, free FAs were turned into respective methyl esters by a reaction with BF_3_ in methanol and analysed on a gas chromatograph equipped with an FID detector (7890A, Agilent, Santa Clara, CA, USA). The C18 capillary column (100 m × 0.25 mm × 0.20 μm, Agilent, Santa Clara, CA, USA) was used for the analyses of FA content. The thermal programme used for the analysis included the following temperature/time sequence: initial temperature of 50 °C, and then a constant rate of increasing by 3 °C/min to reach the final 220 °C. The detector temperature was constant and equalled 270 °C. The presence of every individual FA was checked and identified by the retention times of the respective standard of methylated FA (Sigma-Aldrich).

The extent of oxidation in lipids was analysed by determining the thiobarbituric acid reactive substance (TBARS) content following a slightly modified methodology by Luciano et al. [23]. All meat samples (1.0 g) were thawed after three weeks, and then homogenised with 10 mL of trichloroacetic acid (TCA) to precipitate proteins present in the meat. After centrifugation at 5000× *g* (Sigma 3K30, Sigma Laborzentrifugen GmbH, Osterode am Harz, Germany), the liquid part was passed through Whatman #1 filter paper, followed by the transfer of 2 mL of supernatant to 2 mL of 0.06 M TBA. The reaction mixture was kept in a water bath at 100 °C for 40 min, followed by cooling in an ice-water bath. After immediate chilling to room temperature and centrifugation at 5500× *g*, samples were finally spectrophotometrically analysed (for 15 min, the absorbance was read at 532 nm). The calibration curve was prepared using 1,1,3,3-tetra-ethoxypropane in TCA, as a standard solution. Then, the absorbance of the samples was converted to the mg of malondialdehyde (MDA) present in 1 g of meat, according to a standard curve equation. All analyses were performed in triplicate.

The activity of water (a_w_) in the minced fresh (not frozen) meat samples was measured by Novasina IC-500 W-LAB (Axair Ltd., Lachen, Switzerland). The analyses were carried out in triplicate.

The Hunter scale was used to evaluate the colour of fresh (not frozen) and thermally treated meat samples. Lightness (CIEL L*), redness (a*), and yellowness (b*) were evaluated on the cross-section of muscle fibres in the meat samples using a colorimeter (Minolta CR-400, Konica Minolta Sensing Inc., Osaka, Japan). The results are presented as a mean value calculated from six individual measurements.

In addition, meat marbling, physicochemical characteristics (pH and WHC), sensory traits, texture profile, redox potential, and TBA were evaluated.

Thermally treated meat was also evaluated by its sensory traits, according to the PN-ISO 4121:1998 [24]. The meat was vacuum-packed in PE bags, then, placed in a water bath at 72 °C and incubated until 68 °C was reached in the geometrical centre of the product. Then, the meat was taken out and cut into slices of 1 cm thickness. The prepared samples, after reaching the ambient temperature, were subjected to an evaluation of the organoleptic indicators, such as flavour, taste, colour, consistency, and marbling. Eight trained panellists analysed the samples at room temperature under artificial white light, according to a five-point acceptance scale, from 1 that represented extreme disliking of a sample to 5 that represented extreme liking of a sample [25].

### 2.3. Statistical Analyses

Meat samples (12 samples from each group) were assayed in five replicates for each analysis. A one-factor analysis of variance (ANOVA) was used to assess significant differences between experimental variants as well as the value of the least difference between samples. Variations between mean values in each experimental block were evaluated using the Duncan test with the significance bordering level set at *p* ≤ 0.05 and *p* ≤ 0.01. For all statistical analyses, the programme Statistica 13 (Statsoft Inc., Tulsa, OK, USA) was used [26]. The following experimental model was applied to the study:

y_ij_ = µ + a_i_ + e_ij,_ (1)

where y_ij_ is the value of the observed dependent variable, µ is the value of the population, a_i_ is the influence of the treatment, and e_ij_ is the influence of the random factors.

## 3. Results

The results of the basic chemical composition of the pork meat samples together with the physicochemical traits of the meat are shown in Table 1. All meat samples represented high quality in terms of basic chemical composition and physicochemical parameters. The values of acidity, redox potential, water activity, dry weight, ash, and WHC were at a very similar level without significant intergroup differences. Therefore, it can be stated that different feeding models applied in the study of fattening pigs had no effects on these meat traits. Nevertheless, significant differences were noted in protein (*p* = 0.000) and fat (*p* = 0.027) levels in the meat samples. Loin meat from the pigs in group I showed a greater accumulation of intramuscular fatty components than the loin meat of pigs in group III fed the diet containing 80% W. Meat from groups I and II showed a lower protein level as compared with group III.

The profile of free FA in meat of the fattening pigs is shown in Table 2. Meat collected from animals assigned to group I, receiving dietary 80% HB inclusion, was characterised by the highest content of oleic acid (44.7%) (*p* = 0.049) but at the same time the lowest concentration of palmitic acid (21.5%) as compared with the other two experimental groups (*p* = 0.007).

Table 3 lists the results of the meat colour measurements before and after thermal treatment at 72 °C in a water bath to assure safety of the product. Samples of raw loin obtained from the fattening pigs in group II, which were fed a diet with 40% HB and 40% W, differed significantly from the meat samples of groups I and III meat, with 80% dietary inclusion level of hybrid barley and wheat, respectively. This meat was characterised by a lower lightness (*p* = 0.014) as well as red (*p* = 0.000) and yellow (*p* = 0.000) colour intensity. After thermal processing, meat samples from group III pigs fed the diet containing 80% W were characterised by higher lightness (*p* = 0.007) and lower red colour intensity (*p* = 0.000) as compared with the meat samples of pigs receiving the diet with 80% HB concentrate (group I) or with 40% of each grain (group II).

In Table 4, the results of the cooking losses of meat together with consumer desirability of pork meat are presented. The obtained results indicate that there are no intergroup differences in consumer desirability of the meat samples for the analysed parameters, i.e., flavour, taste, colour, consistency, and marbling. Moreover, significant differences in meat weight losses during thermal treatment as well as meatiness were not observed.

## 4. Discussion

The addition of hybrid barley to the feed of fattening pigs produces similar results for the performance indices as the animals receiving wheat as the basic component of feed [15]. However, in all groups, the feed mixture had a very high nutritional value, the same or higher as compared with the Polish Nutrient Requirements of Swine [14], and no differences in the meatiness were found. Although various feed intakes or energy values of fodder have been shown to affect meat content in dressed carcasses [2,27], the fattening pigs from all studied groups were characterised by a similar body weight (from 119.48 kg to 121.97 kg), slaughter yield (from 73.3% ± 5.28 to 74.3% ± 3.92), and meatiness (from 54.00% ± 0.50 to 54.52% ± 0.35) [15]. These results support the observations previously published by Zhou et al. [28], who indicated that a stable content of lysine in the diet in relation to the metabolisable energy level similarly affected pigs’ growth performances, including meatiness.

The concentration and profile of amino acids present in feed influence muscle development and growth. An increased lysine content in pigs’ fed has been shown to improve the growth of muscle fibres [29]. Wheat grains have a higher level of lysine than barley (3.9 versus 3.7 g·kg^−1^). This could be an explanation for the observed beneficial effects on the carcass characteristics of fattening pigs fed a wheat-based diet [15]. Higher protein and lower fat levels were found in the group III pigs fed a diet with 80% W. The fattening of pigs based on lean growth can be explained by the preference for fast and large accumulation of muscle instead of fat tissue, directly associated with loin deposition [30,31,32]. Turyk et al. [2] showed that pigs fed a diet based on triticale with a higher level of lysine than barley, significantly improved carcass weight, as well as pigs produced loins with larger diameters and less tallow deposition. In addition, a higher content of intramuscular lipids, up to 3.5%, has resulted in significantly higher acceptability of pork by consumers [33]. Many studies have suggested beneficial effects of higher homogenous deposition of fat in meat and its culinary value including juiciness and tenderness of pork [34]. By increasing the level of intramuscular fat, meat juiciness and palatability after heat treatment increased, which in fact improved meat quality [35].

The quality of meat, especially of culinary chunks, as well as meat products relies on raw material characteristics, including fatty tissue colour. The deposition and distribution of lipids in meat, both depend, to a large extent, on feeding composition and freshness, which determine the final colour of the meat. On the one hand, Lampe et al. [6] showed no significant (*p* < 0.05) differences in fat colour between meat from pigs receiving corn or barley. On the other hand, Ringkob [36] presented that the whiter colour perception of pork lipids could reflect freshness of the fat in the opinion of consumers. Therefore, fat colour with higher acceptability by consumers, is obtained after pigs are fed a barley grain diet as compared with a corn-based diet. Moreover, producing pork that contains harder fat but whiter in colour is an advantage from the consumers’ perspectives in the European and Asian markets [37].

The results of this study prove that different feeding models applied in fattening pig nutrition have no significant effects on pork meat quality with respect to acidity, redox potential, content of dry matter, and ash, as well as water activity and WHC. Meanwhile, Joven et al. [38] showed that lower meat juice losses were associated with higher water holding capacity of meat expressing higher than normal pH, measured 24 h post-mortem, which could be explained by a lower glycogen content before slaughter.

The FA profile is determined by breed, animal body weight, and dietary components [10,39]. The chemical composition of pork loin, as well as the FA profile, are relatively stable and less dependent on the dietary lipid types and concentration as compared with the adipose tissue [3]. From a technological point of view, pork with highly saturated fat is more suitable due to the simplicity of handling, processing, and cutability, as well as durability of meat and its products. Therefore, a barley-based diet is more desirable for pigs that produce meat with a whiter and firmer fat tissue. However, among consumers, there are rising concerns about meat and especially meat products that are rich in saturated fats, which is related to an increased risk of developing heart diseases. Since high amounts of SFA are considered to be not healthy for humans, while PUFAs, especially from the n-6 group, create meat lipids that are not hard enough for proper processing, it seems reasonable to increase the content of monounsaturated fatty acids (MUFA) or FA with one double bond (palmitoleic, oleic, and eicosenoic) in meat to compromise the technological quality and consumer preferences.

The FA profile determines meat flavour. Higher levels of SFA and MUFA have an influence on higher acceptability of flavour, juiciness, tenderness, and overall quality, and therefore the likeability of meat [40]. By increasing the content of PUFAs in meat through dietary treatment, fat becomes softer and more acidic, which affects the quality and creates a specific flavour of meat [41].

Many previous studies have shown that the FA profile can be relatively easily modified by using various dietary lipid sources [39,42,43]. The formation of FA incorporated into phospholipids or triglycerides is based on de novo synthesis from dietary carbohydrates or directly from dietary lipids.

Schinckel et al. [44] showed that a typical pig diet based on corn or soybean meal containing a low amount (3–4%) of fats contributed 80% of the triglyceride deposition in meat. Moreover, Daza et al. [45] proved that granulated barley used as the only component in a pig diet, especially during the finishing stage of fattening, increased the content of C18:1n-9 in pork intramuscular fat. Lampe et al. [6] analysed pigs’ backfat and observed a significantly lower concentration of unsaturated FA, including PUFA, while the contents of all SFAs and MUFAs were higher when pigs were fed diets based on barley as compared with corn diets. Contrary results were published by Skelley et al. [46], who studied the FA profile of meat obtained from barley-fed pigs and showed lower MUFA and higher PUFA concentrations in pork as compared with pigs fed a corn-based diet.

In our study, accumulation of the studied FAs in meat followed a similar pattern in all groups, except for palmitic (C16:0) and oleic (C18:1n-9) acids. The total sum of FAs in pork collected from all experimental groups contained more than 40% oleic acid and 20% palmitic acid. Similar levels of FAs were also found in a study by Colonna et al. [47]. In our study, the highest level of oleic acid was noted in the group of fattening pigs fed diets with a high proportion of HB, which could be beneficial from a dietary perspective of pork consumers. However, more than 20% palmitic acid in meat was beneficial from a meat oxidative stability perspective. In group I, which received a diet of 80% HB, the concentration of oleic acid was the highest and the concentration of palmitic acid was the lowest, which is advantageous, since palmitic acid is a SFA while oleic acid is a USFA. This indicates that this meat has more favourable characteristics for a potential consumer.

Fontanillas et al. [48] found that the FA composition of the latissimus dorsi muscle did not exactly reflect the FA content in a diet. The addition of linseed oil into the diet did not significantly increase selected n-3 fat content in meat or in intramuscular fat. Hanczakowski et al. [3] also found that the FA profile analysed in pigs’ longissimus dorsi muscle was not dependent on the FA composition of the fodders. In our study, pork lipid fractions extracted from the longissimus dorsi muscle were varied only in palmitic and stearic acids, when comparing the provided diets.

According to Razmaitė et al. [49], an increase in intramuscular fat content resulted in higher MUFA and lower PUFA contents in most muscles of meat producing animals. With respect to PUFA, Grześkowiak et al. [50] and Jacyno et al. [51] showed that meat collected from lean-type fattening pigs was characterised by a high concentration of PUFA, in particular, linoleic acid. In the present study, the concentration of linoleic acid was higher in meat when barley was used as a feeding component; however, it was not statistically significant, which was an advantage, because, according to Migdał et al. [52], increased linoleic acid concentration worsens the palatability of meat.

The colour of meat is one of the basic criteria in consumer and commercial decision-making during the purchasing process of culinary meat and raw meat products. This parameter is assessed both during the purchase of raw meat and after its thermal processing. Samples of raw loin from the group II fattening pigs fed the diet containing HB and W (40% each) significantly differed from groups I and III (80% dietary inclusion of HB and W, respectively). This meat was characterised before thermal processing by a lower lightness as well as red and yellow colour intensity, which improved the consumers’ acceptance. Sous-vide processing of the samples from all groups resulted in meat with a lighter and less yellow colour intensity with concomitant decreased red colour intensity, which was a standard phenomenon. Meat samples from group III (fed with 80% W) were characterised by higher lightness and lower red colour intensity compared with the meat samples of pigs receiving 80% HB in their diet. The obtained result indicates that HB at an 80% dietary inclusion level has a beneficial effect on the redness of meat (parameter a*) after thermal treatment. It could be assumed that the extraction of pigments was higher from barley than from wheat. (It is known that pigment extraction is higher from husked raw materials than from naked varieties. Colouring compounds are mainly present in the outer layer of grain. However, the carotenoids, which are responsible for the yellow colour of grain, are found in the endosperm [53].)

The obtained results indicated that there were no intergroup differences in consumer desirability of the meat samples. A strong trend towards higher desirability scores with respect to meat flavour and consistency was noted for the meat of the group II animals offered a mixture of hybrid barley and wheat.

Marbling is correlated with intramuscular fat content and deposition. A greater amount of intramuscular fat can contribute to improved juiciness of meat. In our study, no differences in pork loin marbling were observed, which was consistent with a study by Lampe et. al. [6] on the intramuscular fat content in loins from pigs fed a barley-based diet. Moreover, McConnell et al. [54] showed no significant differences in tenderness and marbling of pork loins after feeding pigs barley or corn fodder. Additionally, Lampe et al. [6] reported that adding barley grains to pigs’ feed had no significant effect on pH_24_, sensory evaluated tenderness, and overall desirability, as well as cooking losses. The percentage of barley in pigs’ feed can result in lower flavour as compared with loins cut from carcasses of pigs fed with a mixture of two-thirds yellow and one-third white corn. Spontaneous leakage of meat juice from the loin, especially one designed for exporting, should be minimized due to significantly lower desirability of pork characterised by excessive moisture loss, also when vacuum packaging is applied [55]. According to Lampe et al. [6], replacing corn with barley in pigs’ feed had no significant effect on drip and cooking losses of pork loins kept for 25–27 days after slaughter. In relation to the shelf life of meat, Skelley et al. [46] showed that pork loins could be stored for at least one month, whether the animals were supplied with barley or corn diets.

## 5. Conclusions

Feeds containing 80% hybrid barley can contribute to obtaining meat with high nutritional and dietetic values (increased levels of healthy oleic acid with concomitant reduction of saturated palmitic acid) as compared with a diet of 80% wheat. Pork loin was characterised by similar levels of marbling for all experimental groups, which was closely related to its juiciness and final culinary quality and consumer perceptions of meat. The most favourable colour of meat (reddish) after thermal processing was also noted in the group fed with hybrid barley. Therefore, hybrid barley can be recommended for the diets of fattening pigs, as the only grain ingredient.

## Figures and Tables

**Table 1:** Physicochemical characteristics of meat (loin).

	Treatment			SEM	*p*-Value
	I	II	III		
	HB 80%	HB 40% W 40%	W 80%		
**Nutrients, %**					
Dry Matter	27.41	27.82	27.30	0.078	0.733
Ash	1.62	1.75	1.68	0.081	0.808
**Nutrients as % of Dry Matter**					
Protein	69.11 ^A^	69.94 ^A^	71.74 ^B^	0.194	0.000
Fat	25.51 ^b^	24.15 ^a,b^	22.84 ^a^	0.110	0.027
pH	5.77	5.77	5.78	0.012	0.962
Oxidation potential, µmol TE/100 g	176.11	179.17	187.97	3.690	0.424
Water activity	0.90	0.91	0.90	0.002	0.177
WHC*, %	36.04	36.12	36.14	0.012	0.273

Subscript letters show significant differences between applied treatments analysed in rows: A and B, *p* ≤ 0.01; a and b, *p* ≤ 0.05; WHC*, water holding capacity.

**Table 2:** Fatty acid composition (as % of total FAME*) in meat of the fattening pigs.

FA	Treatment			SEM	*p*-Value
	I	II	III		
	HB 80%	HB 40% W 40%	W 80%		
Palmitic	21.5 ^a^	23.1 ^b^	22.7 ^b^	0.241	0.007
Palmitoleic	3.58	3.50	4.15	0.408	0.803
Stearic	15.1	16.1	15.6	0.208	0.171
Oleic	44.7 ^b^	42.6 ^a^	43.6 ^a,b^	0.358	0.049
Linoleic	10.3	10.3	9.5	0.312	0.578
Eicosenoic	1.01	1.17	1.58	0.173	0.407
Arachidic	3.86	3.26	2.83	0.198	0.097
Saturated (SFA)	36.60 ^a^	39.14 ^b^	38.32 ^b^	0.409	0.021
Monounsaturated (MUFA)	48.28	46.14	47.73	0.516	0.227
Polyunsaturated (PUFA)	15.12	14.72	13.96	0.392	0.503

FAME, fatty acid methyl esters. Statistically significant differences between treatments are indicated by the different subscript letters with the significance level of *p* ≤ 0.05.

**Table 3:** Meat colour parameters.

Colour Parameter	Treatment			SEM	*p*-Value
	I	II	III		
	HB 80%	HB 40% W 40%	W 80%		
**Raw meat**					
L*	48.3 ^b^	46.6 ^a^	48.5 ^b^	0.299	0.014
a**	6.66 ^b^	4.92 ^a^	5.97 ^b^	0.155	0.000
b***	2.58 ^b^	0.90 ^a^	1.97 ^b^	0.131	0.000
**Meat after heat treatment**					
L*	71.7 ^a^	71.4 ^a^	73.0 ^b^	0.219	0.007
a**	3.43 ^a^	3.05 ^a^	2.43 ^b^	0.094	0.000
b***	11.10	11.20	11.00	0.080	0.550

Subscript letters represent statistically significant differences between analysed samples collected from different experimental treatments with the probability of *p* ≤ 0.05. L*, lightness; a**, redness; b***, yellowness.

**Table 4:** Cooking losses, meatiness, and sensory desirability of pork meat after heat treatment.

	Treatment			SEM	*p*-Value
	I	II	III		
	HB 80%	HB 40% W 40%	W 80%		
Cooking losses %	29.93	29.29	29.55	0.350	0.768
Flavour *	3.36	3.57	3.93	0.127	0.182
Taste *	3.79	4.29	3.57	0.145	0.119
Colour *	3.79	3.93	3.93	0.168	0.926
Consistency *	2.86	3.50	3.29	0.179	0.335
Marbling score *	3.14	3.64	3.71	0.142	0.202

* Point scale: from 1, I do not like very much to 5, I like very much.

## Data Availability

The data presented in this study are available on request from the corresponding author.
